# A novel multifunctional oligonucleotide microarray for *Toxoplasma gondii*

**DOI:** 10.1186/1471-2164-11-603

**Published:** 2010-10-25

**Authors:** Amit Bahl, Paul H Davis, Michael Behnke, Florence Dzierszinski, Manjunatha Jagalur, Feng Chen, Dhanasekaran Shanmugam, Michael W White, David Kulp, David S Roos

**Affiliations:** 1 Genomics and Computational Biology, University of Pennsylvania, Philadelphia PA 19104, USA; 2 Department of Biology, University of Nebraska at Omaha, Omaha NE 68182; 3 Department of Veterinary Molecular Biology, Montana State University, Bozeman MT, 59717, USA; 4 Institute of Parasitology, McGill University, Ste. Anne de Bellevue, Quebec H9X 3V9, Canada; 5 Department of Computer Science, University of Massachusetts, Amherst MA, 01003, USA; 6 Department of Biology, University of Pennsylvania, Philadelphia PA 19104, USA; 7 Department of Molecular Microbiology, Washington University School of Medicine, St. Louis MO, 63130, USA; 8 Department of Molecular Medicine, University of South Florida, Tampa FL, 33620, USA

## Abstract

**Background:**

Microarrays are invaluable tools for genome interrogation, SNP detection, and expression analysis, among other applications. Such broad capabilities would be of value to many pathogen research communities, although the development and use of genome-scale microarrays is often a costly undertaking. Therefore, effective methods for reducing unnecessary probes while maintaining or expanding functionality would be relevant to many investigators.

**Results:**

Taking advantage of available genome sequences and annotation for *Toxoplasma gondii *(a pathogenic parasite responsible for illness in immunocompromised individuals) and *Plasmodium falciparum *(a related parasite responsible for severe human malaria), we designed a single oligonucleotide microarray capable of supporting a wide range of applications at relatively low cost, including genome-wide expression profiling for *Toxoplasma*, and single-nucleotide polymorphism (SNP)-based genotyping of both *T. gondii *and *P. falciparum*. Expression profiling of the three clonotypic lineages dominating *T. gondii *populations in North America and Europe provides a first comprehensive view of the parasite transcriptome, revealing that ~49% of all annotated genes are expressed in parasite tachyzoites (the acutely lytic stage responsible for pathogenesis) and 26% of genes are differentially expressed among strains. A novel design utilizing few probes provided high confidence genotyping, used here to resolve recombination points in the clonal progeny of sexual crosses. Recent sequencing of additional *T. gondii *isolates identifies >620 K new SNPs, including ~11 K that intersect with expression profiling probes, yielding additional markers for genotyping studies, and further validating the utility of a combined expression profiling/genotyping array design. Additional applications facilitating SNP and transcript discovery, alternative statistical methods for quantifying gene expression, etc. are also pursued at pilot scale to inform future array designs.

**Conclusions:**

In addition to providing an initial global view of the *T. gondii *transcriptome across major lineages and permitting detailed resolution of recombination points in a historical sexual cross, the multifunctional nature of this array also allowed opportunities to exploit probes for purposes beyond their intended use, enhancing analyses. This array is in widespread use by the *T. gondii *research community, and several aspects of the design strategy are likely to be useful for other pathogens.

## Background

In recent years, annotated genome sequences have become available for many important human and veterinary pathogens, facilitating the exploration of organismal biology. Genome-wide microarrays enable a variety of RNA- and DNA-based queries, contributing to our understanding of genome function and evolution [1,2]. For example, a highly time-resolved expression profiling series through asexual blood stages of the human malaria parasite *Plasmodium falciparum*, using spotted oligonucleotide arrays, revealed a transcriptional program tightly coupled to the cell cycle [3], and further studies have elucidated responses to a variety of drug treatment regimens [4,5]. Higher density photolithographic arrays provide greater resolution of the transcriptional landscape in *P. falciparum*, and have been used to assess genomic variation across multiple isolates [6,7]. A newer generation of tiling arrays and 'next-generation' sequencing is expected to support further applications in gene and SNP discovery, expression profiling, etc. [8]. Such studies have helped to drive research efforts in many areas, including the prioritization of targets for drug, vaccines and diagnostic development [9]. Similar analyses would clearly be valuable for many pathogens, although the development and use of microarrays can be an expensive undertaking.

In order to address the diverse needs of the *Toxoplasma gondii *research community, we have developed a custom Affymetrix array for this protozoan parasite, a prominent source of neurological birth defects during congenital infection, and a cause of encephalitis in immunosuppressed patients. *T. gondii *provides an attractive organism for exploring the utility of mixed use microarrays, for several reasons. First, the parasite genome is relatively small (~65 Mb), and an annotated reference sequence is available [10,11]. Second, a substantial collection of ESTs and SAGE tags from several strains and life cycle stages [12,13] facilitates the assignment of ~8,000 gene models, and provides the basis for validating expression profiling studies. Third, ESTs from multiple strains permits identification of ~3,400 candidate SNPs [14], which have now been validated through additional genome sequencing data that became available in the course of the present study. Fourth, while sexual recombination plays a significant role in generating parasite diversity, including variation in virulence and other important phenotypes [15], *T. gondii *replicates as a haploid, greatly reducing the probe content required for genotyping. Finally, while all of the above characteristics apply to other pathogens as well (including *Plasmodium *spp.), excellent experimental systems are available for *T. gondii *permitting cell and molecular biological studies, forward and reverse genetics, and investigation of host-parasite interactions [16].

Taking advantage of these features, we have designed a novel multifunctional array which enables the following goals: global expression profiling of parasite genes (both nuclear and organellar), and simultaneous analysis of relevant host cell genes; genome-wide high-resolution genotyping; and pilot-scale studies for non-coding regions (promoters, introns, antisense RNAs), alternative expression metrics (exon-level profiling), validation of gene annotation, and polymorphism and transcript discovery. This array also supports inexpensive and efficient genotyping of malaria parasites, based on ~2 K SNPs distributed throughout the *P. falciparum *genome [17,18]. Despite the multifunctional nature of the completed array, low cost and ease of experimental use were maintained, maximizing utility for the broader *T. gondii *and *P. falciparum *research communities.

We have utilized these arrays to provide the first global view of tachyzoite (lytic) stage gene expression for representatives of the three dominant *T. gondii *lineages found in Europe and North America [19,20], greatly increasing our knowledge of gene expression differences [14] between clonotypes. Further, we describe methods for high-resolution genotyping of SNPs from *T. gondii*, enabled by complementing non-redundant genotyping probesets with individual expression profiling probes that intersect SNPs uncovered from recent sequencing of additional *T. gondii *isolates, validating the utility of a combined expression profiling/genotyping array design. Over 5,000 chosen SNPs are used to demonstrate high-resolution mapping of crossover points in the progeny of a historical sexual cross [21]. Additionally, we provide data on select pilot-scale applications, including an exon-level analysis that generally supports the current (mainly computationally predicted) *Toxoplasma *gene models, and SNP discovery in the *T. gondii *plastid (apicoplast).

This report describes the design of this novel multifunctional Affymetrix microarray, and its use for the aforementioned RNA- and DNA-based studies relevant to the biology of *Toxoplasma gondii *and *Plasmodium falciparum*. Table 1 summarizes probe-based design features included on the array, and the following sections provide a brief description of design considerations and selected biological results. Overall, this array incorporates both standard and novel designs, several of which may be relevant to studies on other pathogens or organisms. All data is accessible, and may be queried, via the ToxoDB web site http://toxodb.org.

**Table 1:** Microarray Design^1^.

Application *(for T. gondii unless otherwise indicated)*	# of features	probes/feature	Tiling density	total # probes	% of chip
***Expression Profiling***					
nuclear coding genes (3' biased)^2^	8,058	11		88,638	39.12%
nuclear non-coding genes	22	20		440	0.19%
apicoplast organellar genome (nt)	34,997		25	1,400	0.62%
mitochondrial organellar genome (nt)	6,071		25	243	0.11%
all exons (chr Ib only)^3^	1,080	6		6,480	2.86%
all introns (chr Ib only)	1,080	5		5,400	2.38%
antisense probes (opposite CDS; chr Ib only)	227	20		4,540	2.00%
					
***Gene Discovery***					
ESTs without predicted gene models (nt)	830,867		35	23,739	10.48%
ORFs with BLASTX or TBLASTN hits (nt)	1,263,357		35	36,096	15.93%
					
***Expression Profiling (host species)***					
human (immune response & housekeeping)^4^	301	11		3,311	1.46%
mouse (immune response & housekeeping)^4^	291	11		3,201	1.41%
cat (housekeeping genes)	12		30	360	0.16%
					
***Genotyping***					
*T. gondii *genetic markers	228	40		9,120	4.02%
SNPs inferred from *T. gondii *ESTs, etc	3,490	4		13,960	6.16%
*P. falciparum *genetic markers	1,985	4		7,940	3.50%
					
***Other Analyses***					
SFP discovery on 24 selected genes^5^	23,110		2	11,555	5.10%
promoters (for ChIP) on 12 selected genes^6^	12,000		10	1,200	0.53%
					
***Controls***					
commonly used transgene reporters^7^	39	11		429	0.19%
human & mouse normalization probes				2,200	0.97%
yeast (housekeeping & spike-in probes)				839	0.37%
mismatch probes (genes on chr 1b)	227	11		2,497	1.10%
surrogate mismatch (background) probes				3,000	1.32%

***Total***				226,588	100.00%

**1 **See http://ancillary.toxodb.org/docs/Array-Tutorial.html for a detailed description, including probe sequences.

**2 **A small minority of the 7,793 genes are represented by more than 1 probeset, differing in the degree to which they cross hybridize, while even fewer don't have named probesets of their own as they are interragated by probesets for other genes.

**3 **Non-terminal exons only (terminal exons are interrogated as part of 3'-biased profiling).

**4 **See http://ancillary.toxodb.org/docs/HostResponse.htm for details.

**5 **CDS for AMA1, B1, BSR4/R, GRA3/6/7, MIC2, ROP1/16, SAG1/2/3/4, SRS1/2/9; introns from ATUB, BTUB, BAG1, UPRT. See http://ancillary.toxodb.org/docs/SNPDiscovery.htm for details.

**6 **BAG1, BTUB, LDH1, LDH2, SAG1, SAG2, SAG2C, DHFR-TS, MIC2, GRA1, OWP1, OWP2; see http://ancillary.toxodb.org/docs/ChIP.htm.

**7 **For selectable drug-resistance markers, enzyme and fluorescent protein reporters, etc; see http://ancillary.toxodb.org/docs/TransgeneReporters.seq.

## Results

Probe design and selection required balancing space constraints on the array, a desire to employ standard well-supported experimental methods and analysis algorithms, and new opportunities afforded by custom design. Standard Affymetrix algorithms were used to select probes for traditional applications, including global parasite expression profiling, and genotyping of the several hundred well-characterized genetic markers previously reported for *T. gondii*. This allows for utilization of readily available protocols and software for labeling, hybridization, and analysis. For gene discovery and high-resolution genotyping applications, power analyses suggested that a lower degree of probe redundancy than commonly used in other systems would be sufficient for *T. gondii *and *P. falciparum*, which have relatively small genomes and replicate as haploids. Finally, pilot-scale projects were incorporated to generate preliminary data for several additional applications, including a comparison of methods for transcript profiling and analysis, examination of antisense and intron transcription, chromatin immunoprecipitation studies, expression of selected host genes, and polymorphism detection in highly variable genes.

### Global Parasite Expression Profiling

Expression profiling of the ~8,000 genes identified in the parasite genome (reference strain ME49) is of general interest to the *T. gondii *research community, enabling the correlation of isolate-specific differences in gene expression with differences in virulence, drug sensitivity, differentiation, and other aspects of parasite biology [22,23]. In order to facilitate such experiments, using commonly available reagents and analysis tools, we employed a standard gene expression profiling design, using eleven 3'-biased probes per gene [24]. A perfect match only (PM-only) design was selected, as software supporting such designs is widely available, and exhibits comparable performance to mismatch corrected (PM-MM) schemes across a wide dynamic range [25,26]. The accuracy of expression measures based on PM-only design was confirmed using exogenous spike-in controls, and by PM-MM analysis of genes on chromosome Ib (blue vs. gray in Additional File 1). In addition to profiling the nuclear genome, the mitochondrial and apicoplast genomes were tiled at 25 nt density on alternating strands (using the sequence from strain RH), allowing comprehensive expression analysis for these organellar genomes.

As indicated in Table 1, 7,793 *T. gondii *genes were annotated in the draft 3 nuclear genome sequence, and 3'-biased PM expression profiling probes were designed for all of these genes. In order to evaluate array performance, transcript abundance for *in vitro-*cultivated *T. gondii *tachyzoites was compared with information available from three alternative sources: (*i*) random cDNAs from large-scale unbiased EST sequencing projects [12,27], (*ii*) cDNA abundance inferred by SAGE (serial analysis of gene expression) [13], and (*iii*) a microarray study using spotted clones corresponding to ~500 genes [22]. Because none of these methods was carried out at sufficient depth to identify all transcription units, evaluated transcripts were binned into three groups based on expression level (see Methods). As shown in Additional File 2, this analysis shows good concordance between our array and each of the other three platforms, given our selected binning, over a dynamic range of >100-fold in transcript abundance, indicating reliable performance of the new array.

**Figure 1:**
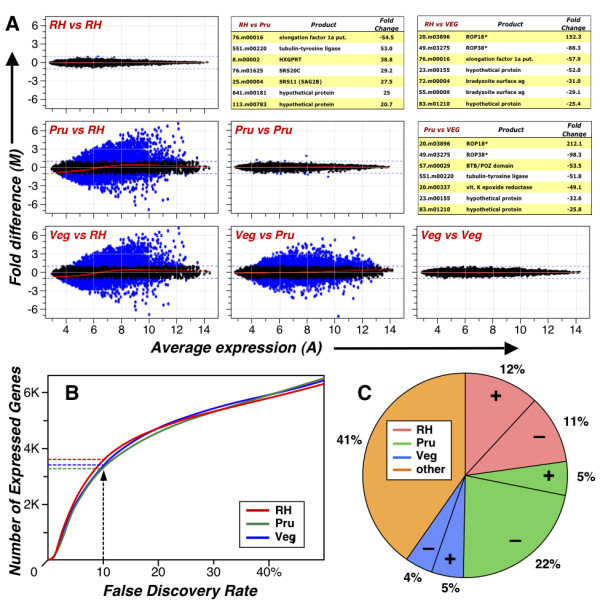
**Differential expression between clonal lineages**. ***A*, **MA plots (intensity ratio versus average intensity) for hybridizations with representatives of the three major clonal lineages of *Toxoplasma *show a very high degree of reproducibility among biological replicates (comparisons shown along diagonal), and a significant number of differentially expressed genes between lineages (blue dots). Tables list genes exhibiting the greatest differences in hybridization intensity for each pairwise comparison, ranked by estimated fold-change (asterisks indicate genes where at least four probes are polymorphic). ***B*, **Gene presence was determined using a 10% false discovery rate (see Materials & Methods for details), resulting in 42% of genes called present in RH-, 38% in Prugniaud-, and 40% in VEG-strain parasites, with an aggregate total of 49% of genes called present in any strain during the tachyzoite (lytic) life stage. ***C*, **5,307 genes exhibit differences in between-strain expression levels at a P-value of 1 × 10^-3 ^(corrected for multiple testing); filtering to eliminate genes for which 4 or more probes are polymorphic, differences in fold change are under 2-fold, or are called absent (at a 10% FDR) leaves 2,078 genes with clear evidence of strain-specific differential expression. The pie chart indicates the distribution of differentially regulated genes by strain (+ and - indicate up- and down-regulation, respectively).

To provide a first global view of expression across the entire *T. gondii *genome, we profiled the rapidly growing lytic tachyzoite stage of three parasite strains (RH = type I; PrugniaudΔHXGPRT (Pru) = type II; VEG = type III), representing the major clonal lineages that define parasite populations and pathogenesis phenotypes in the US and Europe [19,20]. Expression levels were assessed using the Robust Multi-array Average algorithm (RMA; [25]), which summarizes hybridization signals from multiple probes per gene into a single expression value, and present/absent (P/A) calls were made as described in Methods. These P/A results exhibit 83% concordance with calls made by Affymetrix's original MAS5 detection algorithm on chromosome Ib, for which MM probes are available (most differences display very low transcript abundance). As indicated in Table 2 (see also Figure 1B), these studies identified a total 3,986 genes that are expressed in tachyzoite-stage parasites cultivated *in vitro *(49% of the genome at a 10% false discovery rate) -- a significant improvement over the 204 transcripts identified on glass slide arrays (41% of the genes interrogated), in SAGE tag libraries (901), or EST libraries (2,185). Proteomic studies suggest a similar level of expression [28,29].

**Table 2:** Gene Expression in *Toxoplasma gondii*.

	**Number of**:		**Evidence for expression in tachyzoites**:				
	**Probes/Tags**^**1**^	Genes (total = 7793)	**Anystrain**^**2**^	RH (type I)	Pru (type II)	VEG (type III)	No expression
**EST studies**	**125,741**	2,336	2,185 (2,073)	NA	NA	NA	5,608 (5,270)

**SAGE tag studies**	**38,263**	1,229	901 (488)	NA	NA	NA	6,892 (7,305)

**Spotted cDNA arrays**	**2,449**	501	204 (106)	NA	NA	NA	2,245 (2,343)

**Photolithographic array**	**8,058**^**3**^	7,793	3,986 (3,270)	3,395 (1,692)	3,065 (1,472)	3,185 (1,623)	4,072 (2,154)

**1 **Number of *Toxoplasma *ESTs in dbEST, unique SAGE tags in TgSAGEDB, and probesets on the microarrays.

**2 **Values in parentheses reflect a more stringent criteria for evidence of expression: > = 3 SAGE tags or ESTs vs. > = 1; 150% above background vs. > 0% above background; 5% FDR vs. 10% FDR for Affymetrix arrays.

**3 **Some genes have more than 1 associated probeset, differing in degree of potential cross hybridization.

Biological replicates display extremely high concordance across the full range of expression, as shown in Figure 1A. The accompanying tables list genes exhibiting the most highly discordant hybridization patterns in pairwise between-strain comparisons (such queries may also be conducted at ToxoDB.org, using parameters specified by the user). Interestingly, these lists are highly enriched in rhoptry proteins, which are known to play important roles in parasite virulence and pathogenesis [30,31]. Note, however, that many rhoptry proteins are also highly polymorphic, which may in some cases affect hybridization profiles, since expression probes on the array were based on the sequence of type II strain ME49 (asterisks in tables).

Extracting all genes exhibiting differential expression in any pairwise comparison at a P-value of 10^-3 ^(adjusted for multiple testing) yields a total of 5,307 genes (68% of the genome). Further filtering to exclude genes that changed <2-fold, were unexpressed (at a 10% FDR), or were interrogated by a highly polymorphic probeset (defined as those having SNPs in ≥4/11 probes (an empirically determined threshold); see genotyping methods for a description of how polymorphic probes were identified), leaves 2,078 genes displaying statistically significant between-strain differences in expression (26% of all genes). Of these, a single outlier strain could be assigned for 1,239 genes; as indicated in Figure 1C, RH is the outlier in 23%, Pru in 27%, and VEG in 9% of this set. Down-regulation is much more common in Pru (P-value < 2e-16), while no statistical significance is detected with respect to direction of regulation in RH or VEG.

885 high-level biological process Gene Ontology annotations (GO 'slims') were available in the draft 3 *T. gondii *genome annotation, including 677 of the 2,078 genes differentially regulated between strains (Table 3). A hypergeometric test was used to detect over- or under-representation of GO classifications at a significance P-value threshold of 0.05 among expressed genes, or differentially expressed genes between strains. For example, RNA metabolism is statistically over-represented among expressed genes (177 genes expressed, out of 250 annotated in the genome), but most of these are not differentially expressed in tachyzoites cultivated under constant conditions *in vitro*, suggesting that RNA metabolic activity is a relatively conserved or steady function. However, DNA metabolism is under-represented in expressed and differentially expressed genes, while protein metabolism is over-represented in both categories. Analysis of distribution by strain suggests that genes involved in protein metabolism are particularly over-represented in Prugniaud parasites, as are cell cycle genes in VEG, perhaps reflecting the passage history and/or biological phenotypes of these strains.

**Table 3:** Strain-specific differential expression in *Toxoplasma gondii*.

	Number of genes		Significant up- or down-regulation in:				
	**Total**	**Expressed in tachyzoites**^**2**^	**Any strain (*significance***^***3***^**)**	**RH (type I)**	**Pru (type II)**	**VEG (type III)**	**Other pattern**

**All genes in genome**	7,793	3,986	2,078	477	570	193	838

**GO-annotated genes**^**1**^*(process annotations only)*	2,074	1,360	677	143	218	60	256

**Gene-GO Slim mappings**^**1**^	2,764	1,805	885	193	272	80	340

**DNA metabolism**	140	62 *<2e-7*	31 *<0.01*	8	7 *0.03*	4	12

**RNA metabolism**	250	177 0 *.02*	660 *.03*	16	19	4	27

**Protein metabolism**	806	574 *<10e-6*	288 *<0.01*	61	105 *<0.01*	21	101

**Other metabolic process**	528	331	179	31	58	14	76 0 *.04*

**Other biological process**	90	56	32	10 *0.05*	12	0 *0.05*	10

**Transport**	456	297	153	38	36	17	62

**Signal transduction**	85	54	29	7	8	3	11

**Cell cycle/proliferation**	52	28 *0.05*	13	2	5	4 *0.02*	2 *0.04*

**Cell organization/biogenesis**	238	151	70	16	14 *0.02*	10	30

**Cell adhesion**	17	8	2 *0.05*	1	0	1	0

**Stress response**	102	65	22 *0.01*	3	8	2	9

**Unannotated genes**	5,719	2,626	1,401	333	352	133	582

**1 **Multiple GO annotations are available for some genes, and some GO annotations map to multiple GO slim categories (although multiple annotations may also map to the same category). 2,074 genes include 2,979 GO annotations, corresponding to 3,503 GO Slims, for a total of 2,764 gene-GO Slim mappings.

**2 **Assessed using a 10% false discovery rate (see text for details).

**3 **Differential expression determined as described in the text. P-values represent enrichment for GO Slim terms, as determined by the hypergeometric distribution (only values less than 0.05 are shown). Green shading implies under-representation, while yellow shading implies over-representation.

### Global High-Resolution Genotyping

In the field, *T. gondii *populations are characterized by a largely clonal structure, with most strains isolated from North America and Europe falling into one of three dominant clonotypes referred to as types I, II, and III [19,20]. These clonotypes show low intra-lineage polymorphism, but inter-lineage polymorphism of ~1-2%. Variation is dominated by biallelic polymorphisms, and several hundred well-characterized RFLPs, microsatellites, and other markers have been used to map the genetic basis of lineage-specific phenotypes such as virulence [21,30]. Genotyping by RFLP analysis is laborious, providing a bottleneck for mapping studies. We therefore incorporated probes for hybridization-based SNP genotyping onto the microarray, taking advantage of available space left over after the design of probes for expression profiling.

**Figure 2:**
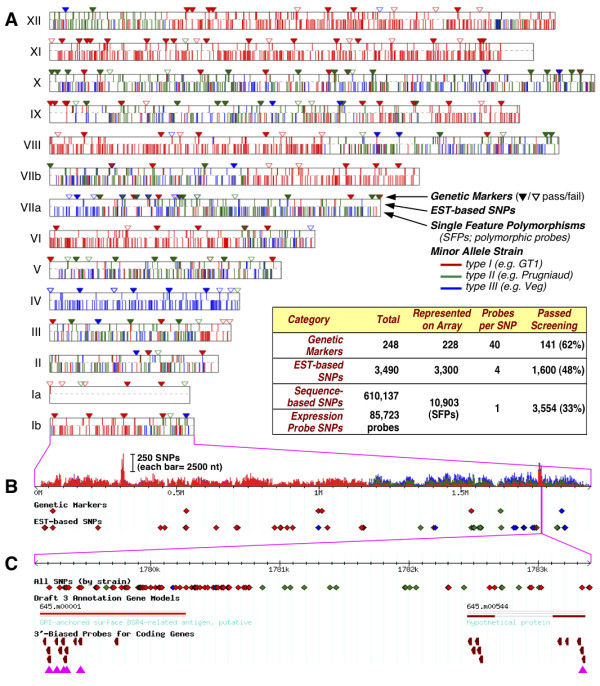
**Genotyping design**. ***A*, **The chromosome map illustrates three tiers of genotyping content present on the *Toxoplasma *microarray. The triangles represent the published RFLP markers, and represent genotyping capabilities prior to this work. The filled triangles represent those markers for which we have provided probesets that passed a rigorous screening process. The top half of each chromosome bar represents the EST-based SNPs, and the bottom half shows the SFPs that have passed screening. The table lists the exact numbers of SNPs represented on the array, those that passed screening, and the probe content for each. ***B***, An expanded view of chromosome Ib indicates the SNP frequency derived from comparative sequence analysis for the three archetypal strains (see Additional File 3), and indicates the location of probesets designed for SNP detection. ***C***, A magnified view of chromosome Ib demonstrating the overlap of SNPs with probes primarily designed for transcriptional profiling. Pink triangles indicate those probes which overlap SNP locations, and can be used to detect SFPs (see text).

Three sets of probes are available for genotyping analysis at increasing resolution, as indicated in Figure 2. 228 of the 248 previously described markers could be mapped to individual SNPs, as indicated by triangles (the remainder were microsatellites or other insertions or deletions not well-suited to genotyping by hybridization). These 228 SNPs were interrogated using standard Affymetrix protocols [32] including 40 probes/SNP: 10 quartets (centered on the SNP, and at ± 1 and ± 4, on both strands), each representing PM and MM probes for both alleles. Consistent with the strategy articulated above, this design enables high confidence genotyping of previously-published markers using off-the-shelf genotyping software.

**Figure 3:**
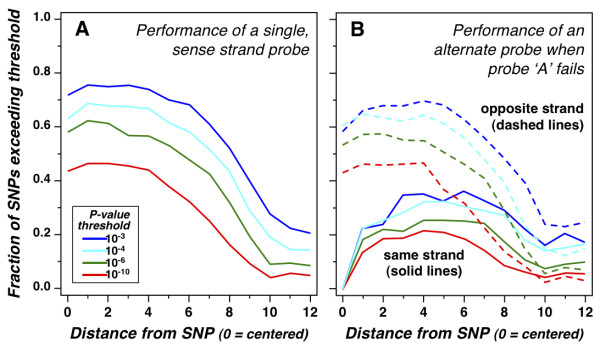
**SNP detection performance**. *A*, Performance of a single, sense strand probe: The ability of a single sense stranded probe overlapping a SNP to call the correct allele as a function of distance from the center of the probe to the SNP is shown. At a stringent P-value threshold of 10^-4^, approximately 65% of SNPs are called correctly using a probe centered exactly on the SNP (see haploid genotyping simulation section in Materials & Methods for a description of P-value calculations). *B*, Performance of an alternate probe when centered sense probe fails: When the centered probe fails to call the correct allele at a chosen threshold, the ability of one additional probe to rescue the call is shown as a function of strand and distance of the probe relative to the SNP. Probes on the sense strand at close distances to the SNP contribute little, presumably due to the same local constraints that caused the centered sense probe to fail, where as the opposite strand centered probe recovers 60% of missed calls at a threshold of 10^-4^. Therefore, at a threshold of 10^-4^, we achieve an 86% success rate.

An additional 3,490 putative polymorphisms were identified based on EST sequences from various parasite strains [14], as indicated by the upper set of vertical bars on chromosomes in Figure 2. The 40 probe approach to genotyping these SNPs would undoubtedly provide a high level of statistical power for distinguishing alleles, but at a high cost, as incorporating all of these probes would necessitate a larger array format (see Table 1), or a genotyping-specific array. Although *T. gondii *is a diploid organism that undergoes meiosis during sexual recombination, mitotic replication occurs as a haploid. As a result, clonal parasite isolates are homozygous at every locus, eliminating the need for heterozygote discrimination. The intuition that haploid genotyping should require fewer probes was confirmed by typing known SNPs using data from the hybridization of (effectively haploid) inbred mouse strains to a densely-tiled resequencing array. As shown in Figure 3A, hybridization of a single PM probe centered on the SNP was able to correctly distinguish between two alleles for >70% of select SNPs in the mouse genome (at a P-value of <10^-3^). Performance falls off with distance from the SNP, dropping below 50% accuracy at ± 8 nt. Performance of adjacent SNPs on the same strand was highly correlated: as shown in Figure 3B, when one probe fails, a probe offset by 1 nt succeeded <25% of the time, and the best performing same-strand probes (offset ± 6 nt) were able to recover SNP detection only ~35% of the time. Similar observations have been made against the *Plasmodium *genome [33]. In contrast, opposite strand probe hybridization was less-highly correlated: when one probe failed, the 0-offset probe from the opposite strand could successfully recover ~60% of SNP calls. As a consequence, the vast majority of SNP detection signal derives from just two probe pairs: one probe for each allele on each strand. A recent study reports similar conclusions using human genotyping data [34]. This four-probe strategy was therefore employed for the 3,490 SNPs inferred from *T. gondii *EST data, and also for probes for 2,000 coding sequence SNPs identified in the *Plasmodium falciparum *genome [35] (Table 1).

The final tier of genotyping design takes advantage of the fortuitous overlap between the 85,723 expression-profiling probes described above and 610,137 SNPs identified by whole genome alignment of three parasite genome sequences (type I strain GT1 and type III strain VEG, in addition to the type II reference strain ME49 (ToxoDB.org); Additional File 3). When these additional genomes became available (subsequent to chip design), it was determined that 10,903 expression profiling probes encompass SNPs. As 11 probes were used per gene for expression studies, and most analysis algorithms are quite robust with respect to individual outliers in the data, these polymorphisms have little impact on expression profiling (data not shown). Such single feature polymorphisms (SFPs; [36,37]) provide a high density set of probes for interrogating polymorphisms, however, albeit at reduced confidence relative to the 4- and 40-probe designs, as they are based on a single probe for a single allele, and not necessarily centered on the SNP (lower vertical bars on chromosomes in Figure 2).

These three classes of SNP analysis probes were screened to remove probesets that failed to consistently yield correct calls across a training sample spanning all three lineages (Additional File 4, and Figure 2, inset). 141 (62%) of the SNPs analyzed using 40 probes (ten quartets) passed this screening (solid triangles in Figure 2). 1,600 (48%) of the SNPs analyzed using the 4 probe strategy (two pairs) passed screening, validating this more-efficient strategy for SNP detection, while 3,554 (33%) of SFP probes passed the filtering step. Note that the percentage of probesets retained is not a measure of accuracy, as excluded probesets usually make no call, rather than calling the incorrect allele. In aggregate, a total of 5,295 typable *T. gondii *genetic markers are represented on the array, and accuracy for those SNPs carried forward is >95%. This corresponds to an average density of 1 SNP per 12 kb genome-wide, representing an ~20-fold increase in resolution over prior genotyping efforts. *Plasmodium falciparum *SNPs were screened using a training set comprised of four strains (3D7, HB3, Dd2, and 7G8), yielding a total of 1,700 SNPs, confirming that the strategy of using two probes per allele is not sensitive to the extreme AT-bias of *Plasmodium *(>80%).

**Figure 4:**
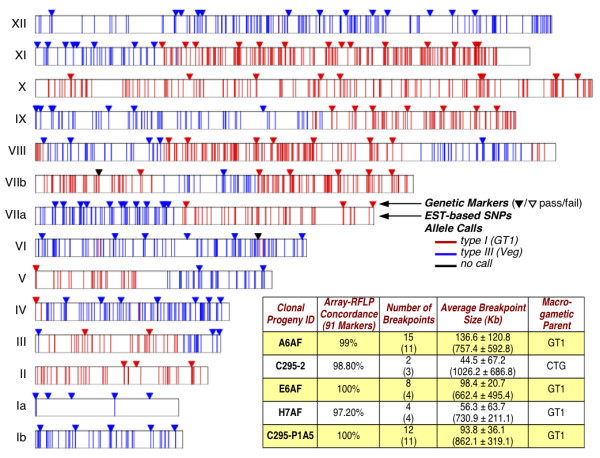
**Detecting crossovers**. A chromosomal SNP map for a recombinant progeny (clone A6AF) of a GT1 (type I) X CTG (type III) cross is represented, along with the published (triangles) and array-based (lines) genotyping calls for this clone. There is almost total agreement between markers called by both methods (>98.5%). The inset table summarizes the benefits of mapping crossovers using the array across 5 randomly selected progeny, showing that on average more breakpoints are discovered, and cover regions that are approximately 11-fold smaller. The numbers in parentheses in the breakpoint columns represent previous results using RFLP analysis.

The complete set of 5,295 typable *Toxoplasma *SNPs was used to genotype recombinant F1 progeny resulting from an experimental cross between the type I GT1 strain and the type III CTG strain [21]. Array-based genotyping identifies distinct cross-over points (red-blue transitions in Figure 4) that are ~99% concordant with the original analysis based on 248 RFLP markers (triangles). The arrays are significantly faster and cheaper, however, and provide much higher resolution. For example, all 11 previously-identified cross-over points in progeny clone A6AF were confirmed, at >5-fold higher resolution. Four additional breakpoints were also discovered (cf. telomeric regions of chromosome VIII, for which no RFLP markers have been defined). In a few cases, individual probes also suggest small cross-overs (cf. red bands in chromosome VI), although these have not been verified experimentally. Similar results were obtained for several other progeny (Figure 4, inset), and characterization of the complete set of available progeny is currently in progress.

In a further illustration of the potential for multiplexing provided by multifunctional chip design, it is interesting to note the potential for using genetic marker probes that fall within coding regions for genotyping in the course of RNA hybridizations. As shown in Additional File 5, reliable calls can be made for ~30 highly expressed polymorphic coding sequence loci that lie close to 3' end of genes. While insufficient for high resolution genotyping, these data provide a useful, inexpensive, first-pass indication of probable genotype, helping to guard against inadvertent strain contamination.

### SNP Discovery (pilot-scale)

Sequence differences distinguishing specific loci have historically been used to discern evolutionary relationships amongst *Toxoplasma *isolates [20]. As an alternative to traditional sequencing, resequencing arrays provide a rapid means for base-calling using DNA hybridization signals. In typical resequencing arrays, a gene is tiled densely with probes, with each PM probe accompanied by the 3 possible MM probes allowing the correct sequence of the target DNA to be determined. Lower density tiling can also be informative (at far lower cost), through the identification of SFPs rather than specific sequence differences. Simulations using mouse resequencing data showed that high performance could be achieved by tiling PM probes only, at 2 bp density. A further (small) boost was observed by alternating the strand of adjacent probes (Additional File 6). As indicated in Table 1 (and Additional File 6, inset), 17 target genes were selected for tiling based on published and unpublished data indicating their utility for strain typing. Several introns were also tiled, in order to determine rates of neutral mutation. In addition, the entire apicoplast genome and a draft mitochondrial genome (assembled from shotgun sequence data and confirmed by PCR) were tiled at 25 bp resolution.

**Figure 5:**
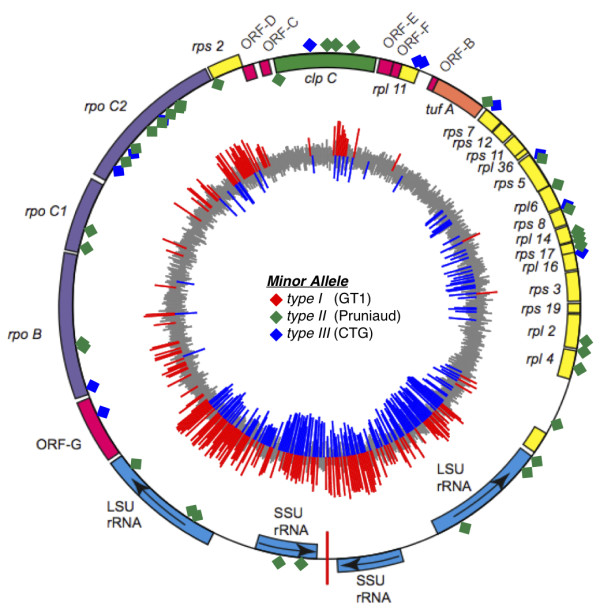
**SNP discovery and gene expression profiling in the apicoplast**. The *T. gondii *plastid (apicoplast; RH strain sequence) was tiled at a 25 nt resolution on alternating strands allowing probe level expression profiling across the entire organelle. Expression patterns (inner circle; red and blue bars represent opposite strands and high absolute expression; grey bars represent low expression levels) are consistent with an operon transcription, with two major origins of transcription evident at the LSU rRNA genes, running in opposite directions (as indicated by the arrows). SFPs were also uncovered using DNA hybridization differences between GT1 (type I), Pru (type II), and CTG (type III), revealing 43 type II SNPs (green diamonds), 12 type III SNPs (blue diamonds), and no type I SNPs (red diamonds).

DNA hybridizations were used to identify apicoplast SFPs, revealing 12 type III SFPs (distinguishing VEG from RH and Prugniaud), 43 type II SFPs (specific to Prugniaud strain parasites), and no type I SFPs, as indicated by diamonds in Figure 5. The paucity of type I SNPs has been reported before [14], and likely represents the sexual history that gave rise to these lineages. These polymorphisms permit the macro-gametic parent to be identified for each of the progeny in the I × III cross shown in Figure 4. As indicated in the table inset, both parental strains produced both macro- and micro-gametes in this cross. RNA hybridizations also support the presumed operon structure inferred from gene organization, although the high degree of variability in adjacent probes remains unexplained (red and blue lines in Figure 5).

### Exon-Level Analysis (pilot-scale)

**Figure 6:**
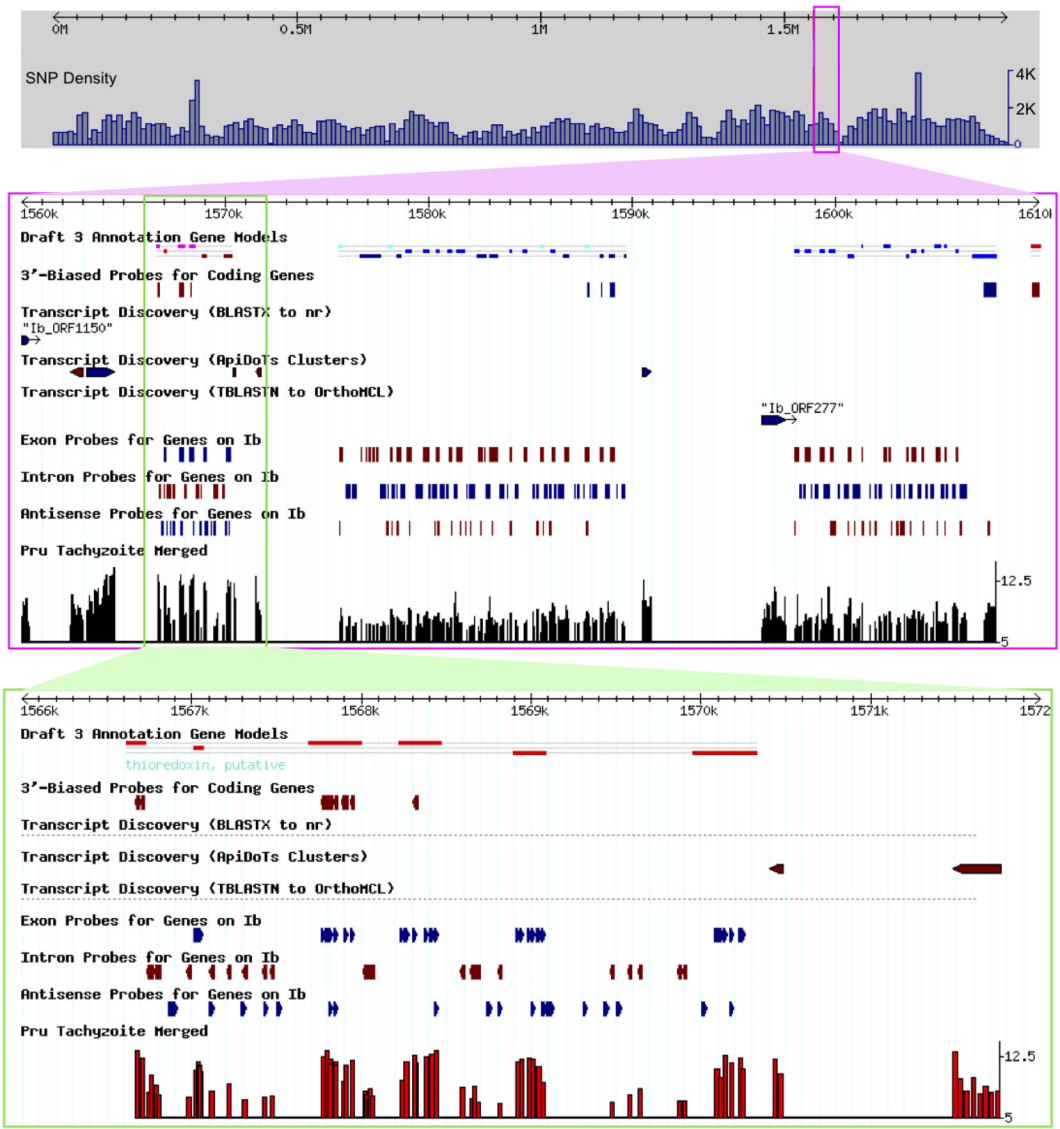
***T. gondii *genes, probes, and probe-level expression profiles**. Top panel shows a 50 kb region of chromosome Ib, illustrating, in addition to the 3'-biased expression profiling probes that are available genome-wide, the high density of probes available for this chromosome, including intron, exon, and antisense probes for each annotated gene (blue genes run from left to right; red from right to left). Transcript discovery probes interrogate unannotated EST clusters (≥3 ESTs) and ORFs (≥150 nt) that intersect with BLAST hits (bitscore ≥100). A barplot provides normalized probe-level expression data (union of sense probe intensities from antisense target kits, antisense probe intensities from sense kit), indicating probable expression of unannotated EST clusters and BLAST hits. See text and Table 1 for further details. Bottom panel displays a 6 kb span at higher resolution, illustrating the validation of gene structure, and comparable transcription levels in upstream ESTs that may correspond to non-coding exons.

Investigating the performance of standard 3'-biased probesets vs. all exon arrays, or antisense expression, would require higher density interrogation than could be justified for a low-cost array. These applications were therefore enabled for a small portion of the genome, allowing the generation of pilot-scale findings to be used in the consideration of future array designs. Chromosome Ib was selected to provide telomere-telomere analysis as it is a single, small (~1.9 Mb) chromosome, exhibiting hundreds of strain polymorphisms (unlike chromosome Ia, which is monomorphic) [38]. Figure 6 illustrates a 50 kb span from chromosome Ib, displaying the variety of probes available for this portion of the genome (in addition to the standard expression-profiling probes available genome-wide), including (*i*) six probes for each exon for every predicted gene, (*ii*) five probes for each predicted intron, and (*iii*) probes to assess the importance of antisense transcription (20 probes from the opposite strand for each of the 227 genes on this chromosome). In addition, probes were tiled at a 10 nt resolution across a ~750 nt span upstream of seven selected genes, enabling pilot-scale chromatin immunoprecipitation studies (not shown). Although not investigated in this paper, the availability of both standard 3'-biased probesets and probes for all exons on chromosome Ib permits a comparison of alternative methods for evaluating gene expression [39]; 3' probes may be less sensitive to differences in mRNA isolation procedures, but aggregate expression signals from every exon might be less sensitive to hybridization artifacts afflicting the 3'-region.

**Figure 7:**
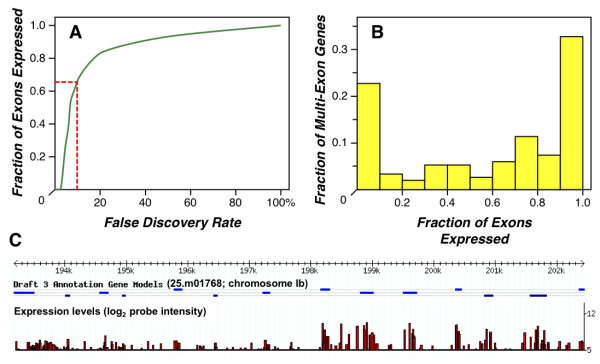
**Validation of gene models**. ***A*, **At a false discovery rate of 10%, ~64% of all exons on chromosome Ib are called "Present" (see Materials & Methods for details). ***B*, **These P/A calls are highly non-random in their distribution, with multi-exon genes showing good consistency among their individual exon calls (i.e. the majority of genes show expression of most annotated exons, or no expression at all). ***C*, **Among genes with inconsistent P/A calls, expression patterns are often clustered, suggesting alternative gene models. For example, the expression patterns associated with the kinesin motor domain-containing protein (25.m01768) suggest a coding start site that begins with the eighth exon.

In addition to providing an alternative measure of expression, exon-level profiling also enables testing of gene model validity, an issue of some concern as ~60% of current *T. gondii *gene models are based solely on computational predictions, without support from experimental evidence such as ESTs or SAGE tags (although proteomics analysis provides additional validation [28,29], and deep sequencing should also help to address this concern). Probe-level analysis often provides a clear distinction between exons and introns. For example, the bottom track in Figure 6 supports the computationally-predicted gene model. Considering chromosome Ib in its entirety, ~64% of all predicted exons were called as present at a 10% false discovery rate (Figure 7A). In addition, as shown in Figure 7B, exon-level 'present' calls are highly consistent within a gene: for genes where at least two exon-level probesets were available, ~33% showed expression of all annotated exons, and 23% showed no expression of any exon (presumably because these genes are not expressed under the experimental conditions employed). Interestingly, among the 44% of multi-exon genes exhibiting discordant expression profiles, exon-level present/absent calls often group together, suggesting alternative gene models, as in the case of the kinesin motor domain-containing protein (25.m01768) shown in Figure 7C, where the first seven predicted exons are not expressed, while the last seven are. It is interesting to note that this interpretation is consistent with a recent study on chromatin marks, which suggest two distinct promoters at this locus [40]. Exon intensities may also suggest differentially spliced transcripts, which have previously been reported in *T. gondii *[41,42].

### Transcript Discovery (pilot-scale)

The current set of *T. gondii *gene annotations represent the results of an algorithm designed to detect consensus gene structures based on several *ab initio *and homology-based gene finders [43], and mapping of an extensive EST library, followed by limited manual curation [44]. Although gene finding has improved in recent years, tiling arrays and deep sequencing have revealed that the level of transcription in most eukaryotic genomes often exceeds what is represented in existing annotation [45,46]. In order to identify promising regions (genome-wide and on either strand) in which to search for unannotated genes, the entire reference *T. gondii *genome was filtered to identify:

(*i*) Unannotated sequences that map to consensus sequences derived by EST clustering (ApiDoTS clusters [27]) containing at least 3 ESTs, yielding 1,189 regions.

(*ii*) Unannotated ORFs with significant BLASTX hits to the non-redundant GenBank database [47], yielding 1,943 intergenic ORFs (≥150 nt) that overlap an HSP (bitscore ≥100) by more than 100 nucleotides.

(*iii*) Unannotated ORFs (≥150 nt) that are significant matches (overlap ≥100 nt, bitscore ≥200) for the query set of OrthoMCL ortholog database sequences using TBLASTN, resulting in 450 ORFs.

Statistical analysis of human spike-in data suggested that a tiling density of 35 bp would be sufficient to reliably detect missed exons across a useful dynamic range of target concentrations (Additional File 7). The 50 kb span displayed in Figure 6 shows moderate expression associated with an unannotated ORF (Ib_ORF1150) that hits a hypothetical protein in *Plasmodium yoelli *(PY00596), and two adjacent unannotated EST clusters exhibiting high expression.

### Host Expression Profiling (pilot-scale)

Most of the intended applications for this microarray focus on the biology of *T. gondii*, but parasite pathogenesis clearly involves alterations in host cell/organism expression as well. Several key players involved in host adaptive and innate immune responses to *T. gondii *have been reported [48], and a small-scale transcriptional profiling study identified additional genes [49], but the complete host transcriptional profile during *T. gondii *infection is unknown. In order to permit evaluation of host immune responses, we included both human and the corresponding mouse orthologs (NCBI HomoloGene) for 260 host genes on the array, representing a comprehensive set of cytokines, chemokines, receptors, and other genes likely to function at the parasite-host interface (see Additional File 8 for a complete list). PM-only probesets were derived from the human U133Plus 2.0 and mouse 430 2.0 arrays [50,51]. This collection provides parasitology researchers with an economical opportunity for studies that may not require genome-wide expression profiling, and the opportunity to explore expression changes in both host and pathogen in parallel. To permit unambiguous detection of signals from parasite vs. host mRNA, parasite gene expression probes were pruned to minimize the potential for cross-hybridization to human or mouse mRNA sequences, and preliminary analysis indicates essentially no reduction in specific signal when a 100-fold excess of host RNA was included in parasite expression profiling studies (data not shown).

## Discussion

This report describes a multifunctional microarray supporting a wide range of studies on the protozoan parasite *Toxoplasma gondii *(Table 1). Expression profiling confirms previous results obtained on various platforms (Additional File 2), allowing analysis to be extended genome-wide (Figure 1). The perfect match only design employed for this array compares favorably with a small-scale analysis including mismatch controls (for chromosome Ib only), and preliminary results indicate that small differences in sensitivity at low expression levels can be restored using a pool of surrogate mismatch probes selected on the basis of nucleotide composition (Additional File 1). Exon-level analysis (Figure 7), generally support the overall accuracy of *T. gondii *gene models. Tiling of regions with significant BLAST or EST hits, but no current gene call, allow the interrogation of additional transcriptionally active regions (Figure 6). All of the expression profiling data described in this report has been deposited with NCBI's **Gene Expression Omnibus **(GEO), and loaded into ToxoDB.org [11], enabling a wide-range of queries. For example, users may wish to compare genes identified by EST, proteomics, chromatin immunoprecipitation, and microarray analysis. The availability of whole genome expression profiling arrays is expected to facilitate a wide range of studies on stage-specific expression, mutant characterization, etc.

Comparative analysis of expression levels in representatives from each of the three lineages that dominate *T. gondii *populations in the US and Europe [15,20] shows that ~49% of the 7,793 *T. gondii *genes identified in draft 3 annotation are expressed in tachyzoite-stage parasites (Table 2), implying that approximately half of the genome may function exclusively in the latent or sexual life stages. As demonstrated for tachyzoite transcriptional profiling, the microarray described in this paper can identify and prioritize genes that play key roles in these other life stages for further functional studies. For example, tachyzoite-to-bradyzoite stage transition experiments have yielded a robust set of genes that appear to be involved in early bradyzoite differentiation (Roos et. al., manuscript in preparation).

It is interesting to note the unusually low variance observed in biological replicates (Figure 1A), perhaps reflecting the homogeneity of the intracellular niche occupied by these parasites. These studies also reveal substantial differential expression between lineages (Table 3), with ~26% of expressed genes showing significant differences in at least one strain (although the high percentage of differentially expressed genes may simply reflect the low variance among biological replicates, which raises statistical power to detect subtle changes in expression levels). Secreted proteins known to play an important role in virulence [30,31] are particularly notable for their extreme inter-strain differences in gene expression (Figure 1A). ToxoDB employs strain-specific library files (a mapping of probes to genes) that eliminate polymorphic probes to avoid false positives due to SNPs in determining differential expression.

The most unusual aspect of this study is the incorporation of both expression profiling and genotyping probes on the same array, broadening the utility of this chip for biological analysis. Standard array-based genotyping strategies (40 probes) were modified in light of the discovery that same-strand probes are largely redundant (Figure 3), particularly for haploid *T. gondii *parasites. Probesets including only 4 features passed high-stringency screening nearly as frequently as 40 feature probesets (62% vs. 48%), but are much more economical, improving *T. gondii *genotyping from the >300 kb resolution currently available using 186 RFLP markers [52] to ~37 kb (using 1,600 markers; Figure 2). 2,000 well-validated *P. falciparum *SNPs were also included on this array, providing an economical means for genotyping of the most lethal human malaria parasite [17,18].

Sequences for two additional *T. gondii *isolates were released subsequent to chip production, revealing >600 K biallelic SNPs (Additional File 3). 33% of expression profiling probes that fortuitously overlap SNPs passed the strict quality control parameters established for genotyping, despite the absence of a complete probe quartet centered on the SNP, providing an additional ~3,500 reliable genotyping markers (Figure 2, inset). Using the entire set of 5,295 typable markers to evaluate the progeny of a cross previously analyzed by standard methods [21] revealed ~99% concordance, while mapping crossovers to higher resolution and identifying several additional recombination points (Figure 4). It will be interesting to investigate the several instances of apparent micro- and telomeric crossovers identified in this analysis. Higher resolution genotyping at lower cost should greatly facilitate QTL and other genetic mapping studies [21,52].

Numerous other features were included on this multifunctional array (Table 1), including surrogate mismatch probes to facilitate background subtraction (Additional File 1), and probes for array-CGH studies on the tiled apicoplast genome. Apicoplast SFPs were used to demonstrate uniparental inheritance, with either parent able to provide the macrogamete (Figure 4, inset); transcript profiling (Figure 5) supports the proposed operon model for transcription of this organellar genome [53].

## Conclusions

The driving motivation for this array design was to support low cost whole genome expression profiling for the protozoan parasite *Toxoplasma gondii*, by reducing standard chip size (in accordance with the relatively small parasite genome), and eliminating mismatch probes (which provide minimal advantage). This reagent has been widely adopted by the *T. gondii *research community. Excess space available on the array was exploited to support high resolution, low cost genotyping, taking advantage of the discovery that 4 feature probesets are nearly as effective as 40 feature probesets. The multifunctional nature of this array has provided many unexpected advantages, including the opportunities to exploit expression profiling probes as SFP markers, and the ability to use genotyping probes for strain validation during RNA hybridization experiments. Many of the principles employed in this design are applicable to other species.

## Methods

### Array Design and Production

*T. gondii *genome sequences (type II strain ME49) and gene models (draft III) were obtained from ToxoDB.org. The apicoplast genome sequence (type I strain RH) was from GenBank (acc# NC001799) and the mitochondrial genome sequence was inferred by alignment of sequence fragments (D. Shanmugam and L. Peixoto, unpublished data). *P. falciparum *SNPs were kindly provided by X. Su [35]. A custom photolithographic microarray containing 25-mer oligonucleotides (11 micron feature size, 169 format) was designed and manufactured using the Affymetrix CustomExpress™ Array Program (Santa Clara, CA). Content is described in Table 1, and arrays are available through the Penn Microarray Facility. For further information, including custom analysis algorithms, library files, and ordering instructions, visit ToxoDB http://www.ToxoDB.org.

### Expression Profiling Hybridizations and Data Analysis

For expression analysis based on the 3'-biased probesets, PrugniaudΔHXGPRT, RH, and VEG strain parasites were cultured in human foreskin fibroblast (HFF) cells as previously described [16]. Prior to host cell rupture, cells were scraped from the flask and spun at 300 g for 9 min. The resultant pellet was lysed with Buffer RLT from the Qiagen RNeasy Mini Kit (Valencia, CA) and RNA was extracted according to the manufacturer's instructions. Labeled cRNA was created using the One-Cycle Labeling protocol in the Affymetrix GeneChip^® ^IVT Labeling Kit (Santa Clara, CA). RNA used for exon-level analysis was isolated from Prugniaud strain parasites using the same procedures, but labeling was performed with the Affymetrix Whole Transcript Sense Target Labeling Kit according to manufacturer's instructions without rRNA reduction. Hybridization, washing, and scanning of arrays was performed using standard Affymetrix instrumentation and protocols for 11 micron, 169 format arrays. Biological triplicates were generated for each strain and expression values computed using the RMA implementation (default parameters) in the affy package from Bioconductor [54]. Differential expression was determined using SAM at a 1% false discovery rate. The data from these 12 hybridizations have been deposited into GEO [GSE20145]. A more inclusive transcriptomic comparison set was subsequently generated from additional *Toxoplasma *strains, which serves as the data source for Figure 1A and are publicly available for querying and download from ToxoDB.org.

### Comparison to SAGE, EST, and glass array data

38,263 unique 3-prime *T. gondii *SAGE tags [13] were associated with gene models if they mapped (exact 14-mer match) within a predicted CDS or the downstream 700 bp region (estimate of 3^rd ^quartile of UTR-length distribution based on UniGene cluster analysis done for this study), resulting in 1,229 genes being linked with SAGE data. The 125,741 *T. gondii *ESTs deposited in dbEST (NCBI) were mapped to the reference genome using Splign [55] and EST-gene links made by filtering for EST coverage (≥80%) and extent of overlap (≥50 nt) with genes and their estimated 3'-UTR regions. This resulted in 2,336 genes being associated with EST data. Glass array data [22] for 2,449 sequenced cDNA spots was associated with genes using the same criteria as for ESTs, linking 501 genes with these hybridization intensities. In total, 3,077 genes (40% of the genome) were linked to at least one of these three sources of expression data. For comparison with parasite tachyzoite expression profiles derived from the Affymetrix array, tag counts from EST and SAGE data were filtered to include only unbiased (i.e. not normalized) tachyzoite stage libraries (SAGE: day6, MSJ, RH, and B7), and then normalized to tags per 100,000 (Tp100K = observed count * (100,000/total tachyzoite tags). The resulting tag values were binned to make expression level calls (low, medium, high) based on the following thresholds: low expression when ≤0.02% of cellular mRNA content (i.e. 0 < Tp100K <20); medium expression from 0.02 - 0.1% of mRNA content (20 ≤Tp100K < 100); high expression = ≥ 0.1% mRNA (Tp100K ≥ 100). Data from the glass arrays (tachyzoite controls from a differentiation experiment) was binned as follows: a gene was defined as exhibiting low expression level if hybridization was indistinguishable from background, medium if up to 2× background, and high if >2× background. See inset in Additional File 2 for relative numbers of genes exhibiting high, medium, and low level expression by each method.

### Present/Absent Calls for Genes and Exons

For gene-level "present" calls, labeled sense-strand mRNA was used to define a null background RMA distribution for each gene using the 3'-biased probesets. P-values for presence were then assigned to each gene based on hybridizations with labeled antisense RNA. Using the Benjimini-Hochberg method these P-values were used to set a 10% FDR threshold. Exon-level presence calls were made using a similar procedure, with the null distribution of RMA values defined using antisense RNA, as exon probes are antisense in orientation to their corresponding mRNA. A 10% FDR was used for calling exon presence.

### Simulations of Haploid Genotyping and SNP Discovery

DNA from each of the inbred mice DBA/2J and C57/B6 were hybridized to a 1 bp resequencing microarray designed for random regions of the C57/B6 mouse genome and 109 high confidence SNPs were identified using standard resequencing analysis algorithms (D. Kulp, unpublished results). Using this data as a reference for the detection of known SNPs, P-values associated with a Kolmogorov-Smirnov test distinguishing the two strains were computed using different probe tiling strategies.

### Genome alignments and SFP Discovery

Genomic assemblies of GT1 (type I) and VEG (type III) were obtained from ToxoDB.org and aligned to the reference strain ME49 (type II) using NUCmer [56]. Regions of ME49 with unambiguous mappings were scanned for SNPs using the show-snps program from the MUMmer package. 10,903 single feature polymorphisms (SFPs) were uncovered by searching for predicted SNPs that overlapped one of the 85,723 3-prime biased probes designed for expression profiling. Apicoplast SFPs were discovered using hybridization differences among apicoplast probes between GT1 (type I), Pru (type II), and CTG (type III) DNA hybridizations.

### Genotyping Hybridizations and Data Analysis

*Toxoplasma gondii *genomic DNA was isolated from RH, Pru, VEG, and select recombinant progeny via scraping and pelleting cultured parasites (as above) and then using the Gentra Systems Generation DNA isolation kit (Minneapolis, MN) according to the manufacturer's instructions. Purified DNA was diluted in 750 ul TE pH 8.0 containing 10% glycerol with the addition of 2 ul molecular biology-grade glycogen (20 mg/ml). Approximately 800 ng of diluted DNA was added to an Invitrogen nucleic acid nebulizer on ice, and compressed nitrogen was used at 40 psi for 3 min to shear the DNA. Fragmented DNA was alcohol-precipitated, heated, and labeled for 2 hr using the Invitrogen BioPrime Array CGH Genomic Labeling module with biotin-14-dCTP according to the protocol from the manufacturer. Labeled DNA was cleaned with the Purification module and hybridized to the microarray as described above. Data analysis was conducted in Bioconductor, using custom R algorithms. Genetic markers were called using the Wilcoxon sign rank test using the 10 PM probe intensities for allele 1 versus the 10 PM probe intensities for allele 2 (P-value ≤.10). EST-based SNPs were called on the basis of the mean allelic ratio of the 2 pairs of PM probes (ratio ≥ 1.5). SFP calls were made based on the distances in the background corrected (RMA) and normalized (quantile) intensity value of a polymorphic probe in a progeny hybridization to its counterparts in parental hybridizations.

## List of Abbreviations used

HXGPRT: hypoxanthine-xanthine-guanine phosphoribosyl transferase; RMA: robust multi-array average; SFP: single feature polymorphism; SNP: single nucleotide polymorphism; PM: perfect match;

## Authors' contributions

AB, DK, MJ, and DSR conceived and participated in the design of the platform; PHD, MB, FD, and DS carried out wet experiments. AB, PHD, MB, FC, MWW, and DSR conducted the analysis of chip data. The manuscript was drafted by AB, PHD, and DSR. The final version was read and approved by all authors.

## Supplementary Material

Additional file 1 **Comparison of background correction methods**. The mismatch (MM) probes included for all genes on chromosome Ib, and 3,000 surrogate mismatch probes (SMM), allows comparison of PM-only (RMA v2; blue), PM-MM (grey), and PM-SMM (red) methods for background correction (Prugniaud strain RNA). Background correction using the SMM probes was determined by subtracting the trimmed mean of all surrogate probes with matching GC content. Different PM-SMM trajectories observed at low PM intensity reflect increased hybridization background in high GC content probes (inset). Over most of the dynamic range, all three methods yield similar results, although PM-MM tends to attenuate signal as MM probes capture true signal in addition to background. PM-only methods may lose some sensitivity at the lower end of the dynamic range relative to either SMM or MM-corrected methods, which appear to be comparable in their performance. Click here for file

Additional file 2 **Validation of *Toxoplasma *gene chip expression values**. *T. gondii *SAGE, EST, and glass array data was mapped onto gene models, and binned abundance calls (high, medium, low) for the lytic (tachyzoite) stage were made for an aggregate total of 3,077 genes, as described under Materials & Methods. Expression values for Prugniaud-strain tachyzoites determined using the photolithographic oligonucleotide microarray described in this report are highly concordant with results from all other platforms, with an average median difference between successive bins of ~4-fold. Horizontal black bars indicate median values; boxes show lower and upper quartiles. Click here for file

Additional file 3 ***Toxoplasma *SNP Map**. Whole genome alignments of representatives of the three main clonal lineages (strains GT1, ME49, and VEG) were used to uncover biallelic SNPs. Each discovered SNP is classified as type I, II, or III, referring to the strain that contains the minor allele. Each stacked bar represents the SNP type counts in a 2,500 nt non-overlapping bin. The vertical distance between chromosomes corresponds to 250 SNPs. Click here for file

Additional file 4 **Screening SNPs**. SNPs were screened for predictable behavior using hybridizations with RH-, Prugniaud-, and VEG-strain parasites. ***A*, **141 genetic markers (61%) resulted in correct allele calls (P-value < .1) in all 3 screening hybridizations. ***B*, **1,600 EST-based SNPs (46%) were carried forward after screening (allelic ratio threshold >1.5). ***C*, **90% of *P. falciparum *SNPs are called correctly (allelic ratio threshold >1.5). ***D*, **3,554 SFPs (33%) passed filtering based on their behavior in pairwise comparisons in the three screening hybridizations. For example, type I SFPs (polymorphic probes containing a type I SNP) that were carried forward had significantly suppressed probe intensities in type I vs. type II or type III comparisons, but displayed no significant difference in a type II vs. type III comparison. Click here for file

Additional file 5 **Multiplexing experiments**. The ability to reliably differentiate alleles of RFLP genetic markers that fall within coding regions using RNA hybridization data is illustrated (i.e. genotyping analysis as described in the Methods section applied to RNA hybridizations). For example, the type I RH strain correctly exhibits high relative minor allele strength (minor allele/(major allele + minor allele)) for most type I SNPs, but not for type II or type II. In addition, miscall rates are very low when the marker is close to the 3-prime end of the gene, but rise appreciably after ~1000 bp. Click here for file

Additional file 6 **Tiling density for SNP discovery**. The ability to detect known homozygous mouse SNPs decreases with increasing distance between the centers of successive probes, as illustrated by the area under the curve (AUC) of the ROC measurements derived from a custom SNP classifier applied to each gap size. A 2-bp tiling strategy, with adjacent probes on alternate strands, offers near perfect SNP detection. The inset table lists the genomic loci that were tiled. Click here for file

Additional file 7 **Probe density for exon-level analysis**. HGU95 spike-in data (Affymetrix) was used to test the effects of decreasing probe number on present/absent calls using the MAS5 algorithm. Five probes offer reliable transcript detection across a dynamic range ≥8 pM; as the median exon size in *T. gondii *is 171 bp (inset), a tiling density of 35 bp was selected for exon discovery probes. In order to err on the side of conservatism, six probes were selected for the 'all exon' probesets on chromosome Ib. Click here for file

Additional file 8 **Human and mouse genes included on the array**. The table describes human and mouse probesets available on commercial Affymetrix arrays that were included on the *T. gondii *microarray. Click here for file
