# Supplementary figures and images for: A novel multifunctional oligonucleotide microarray for Toxoplasma gondii
Source: BMC Genomics. 2010 Oct 25;11:603. doi: 10.1186/1471-2164-11-603 (PMC3017859; doi:10.1186/1471-2164-11-603)

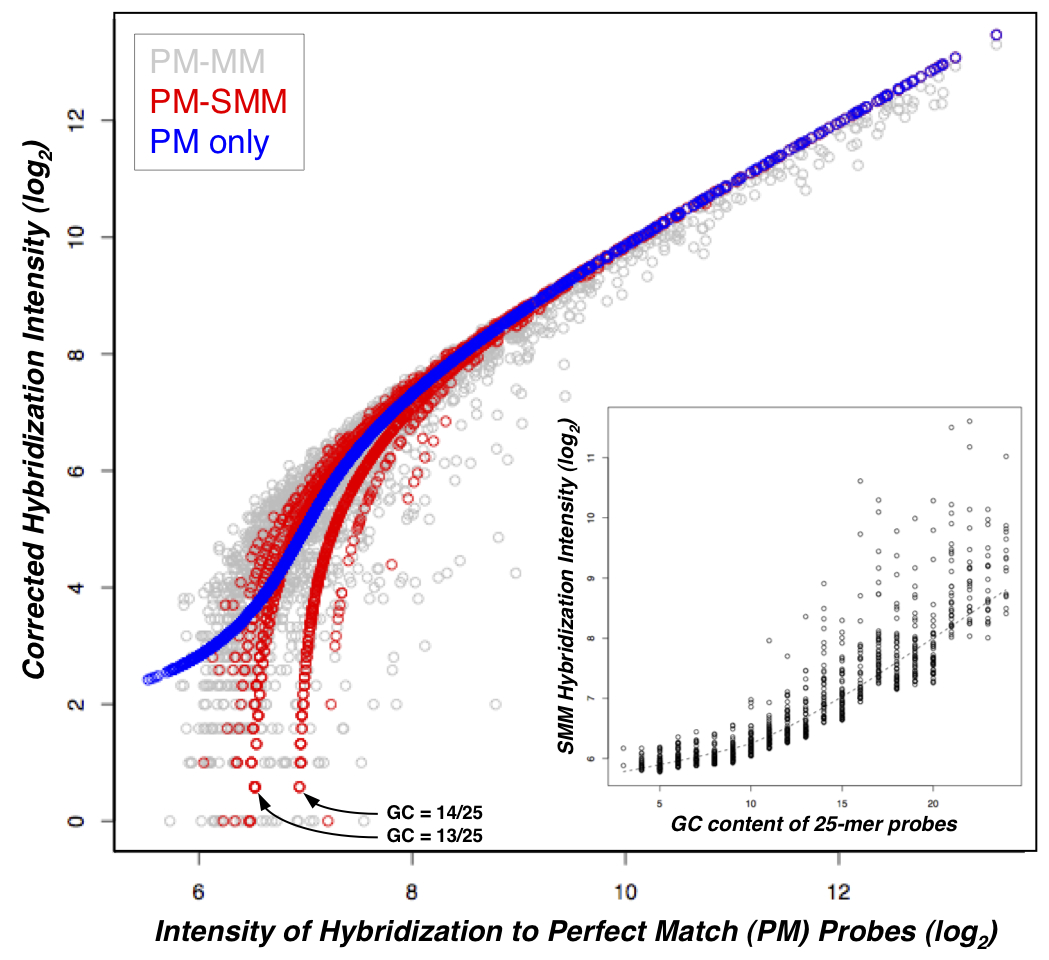

Supplement: Additional file 1 — Comparison of background correction methods. The mismatch (MM) probes included for all genes on chromosome Ib, and 3,000 surrogate mismatch probes (SMM), allows comparison of PM-only (RMA v2; blue), PM-MM (grey), and PM-SMM (red) methods for background correction (Prugniaud strain RNA). Background correction using the SMM probes was determined by subtracting the trimmed mean of all surrogate probes with matching GC content. Different PM-SMM trajectories observed at low PM intensity reflect increased hybridization background in high GC content probes (inset). Over most of the dynamic range, all three methods yield similar results, although PM-MM tends to attenuate signal as MM probes capture true signal in addition to background. PM-only methods may lose some sensitivity at the lower end of the dynamic range relative to either SMM or MM-corrected methods, which appear to be comparable in their performance. [file 1471-2164-11-603-S1.JPEG]

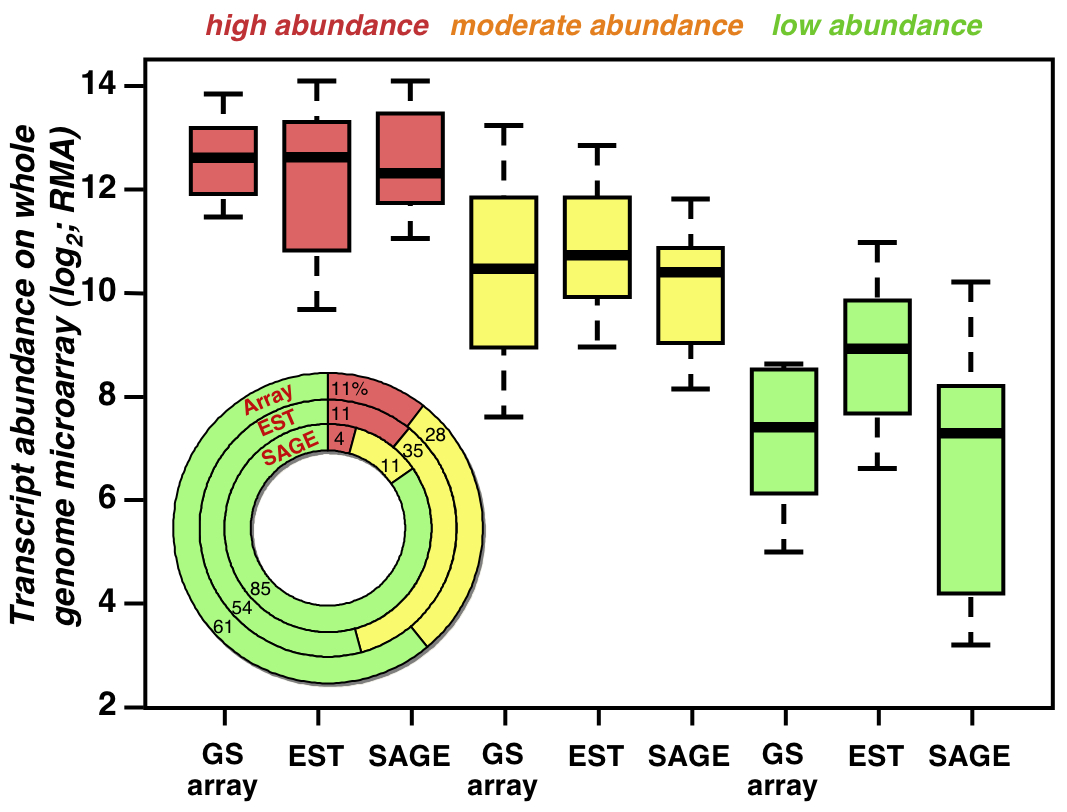

Supplement: Additional file 2 — Validation of Toxoplasma gene chip expression values. T. gondii SAGE, EST, and glass array data was mapped onto gene models, and binned abundance calls (high, medium, low) for the lytic (tachyzoite) stage were made for an aggregate total of 3,077 genes, as described under Materials & Methods. Expression values for Prugniaud-strain tachyzoites determined using the photolithographic oligonucleotide microarray described in this report are highly concordant with results from all other platforms, with an average median difference between successive bins of ~4-fold. Horizontal black bars indicate median values; boxes show lower and upper quartiles. [file 1471-2164-11-603-S2.JPEG]

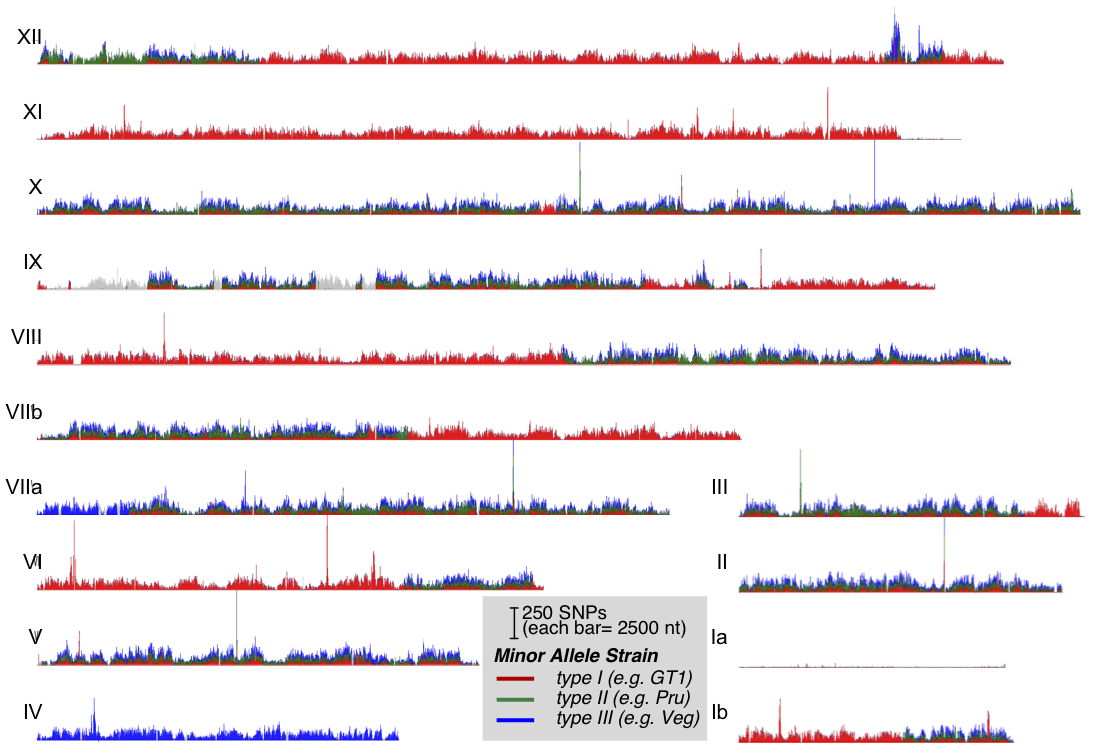

Supplement: Additional file 3 — Toxoplasma SNP Map. Whole genome alignments of representatives of the three main clonal lineages (strains GT1, ME49, and VEG) were used to uncover biallelic SNPs. Each discovered SNP is classified as type I, II, or III, referring to the strain that contains the minor allele. Each stacked bar represents the SNP type counts in a 2,500 nt non-overlapping bin. The vertical distance between chromosomes corresponds to 250 SNPs. [file 1471-2164-11-603-S3.JPEG]

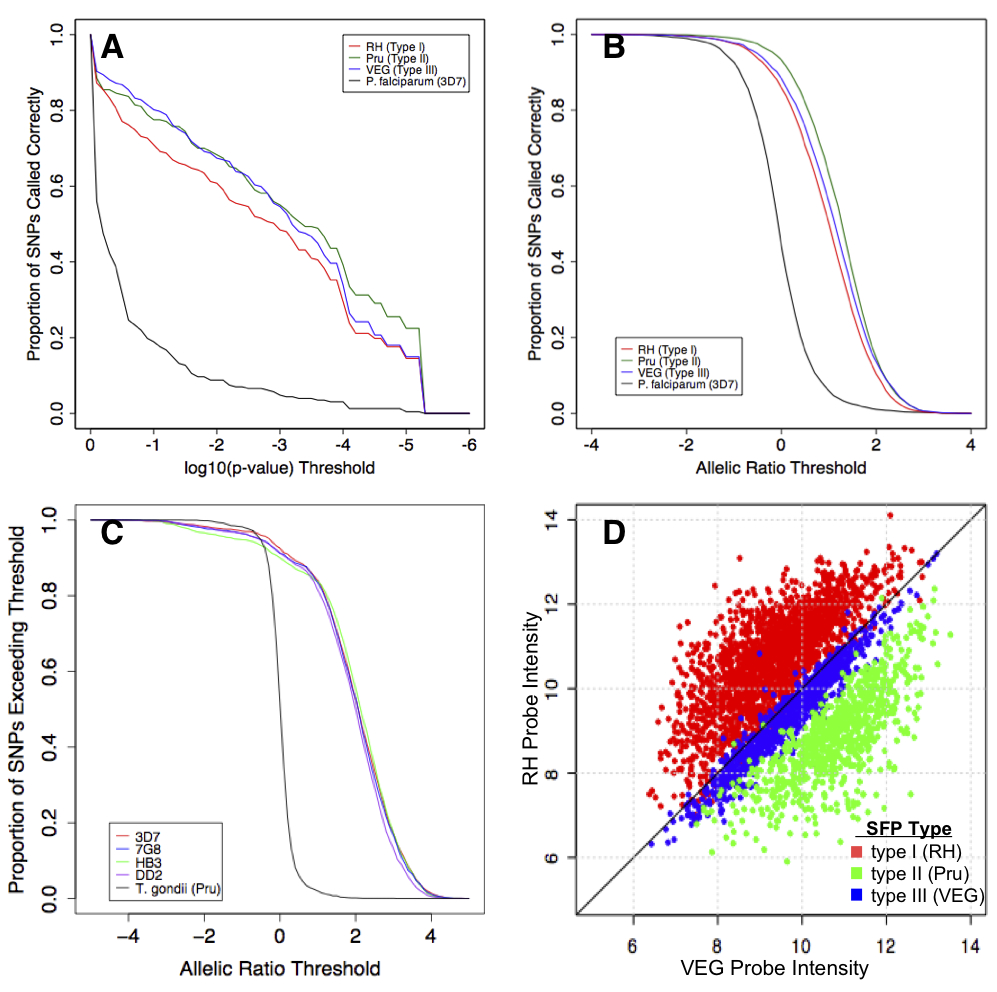

Supplement: Additional file 4 — Screening SNPs. SNPs were screened for predictable behavior using hybridizations with RH-, Prugniaud-, and VEG-strain parasites. A, 141 genetic markers (61%) resulted in correct allele calls (P-value < .1) in all 3 screening hybridizations. B, 1,600 EST-based SNPs (46%) were carried forward after screening (allelic ratio threshold >1.5). C, 90% of P. falciparum SNPs are called correctly (allelic ratio threshold >1.5). D, 3,554 SFPs (33%) passed filtering based on their behavior in pairwise comparisons in the three screening hybridizations. For example, type I SFPs (polymorphic probes containing a type I SNP) that were carried forward had significantly suppressed probe intensities in type I vs. type II or type III comparisons, but displayed no significant difference in a type II vs. type III comparison. [file 1471-2164-11-603-S4.JPEG]

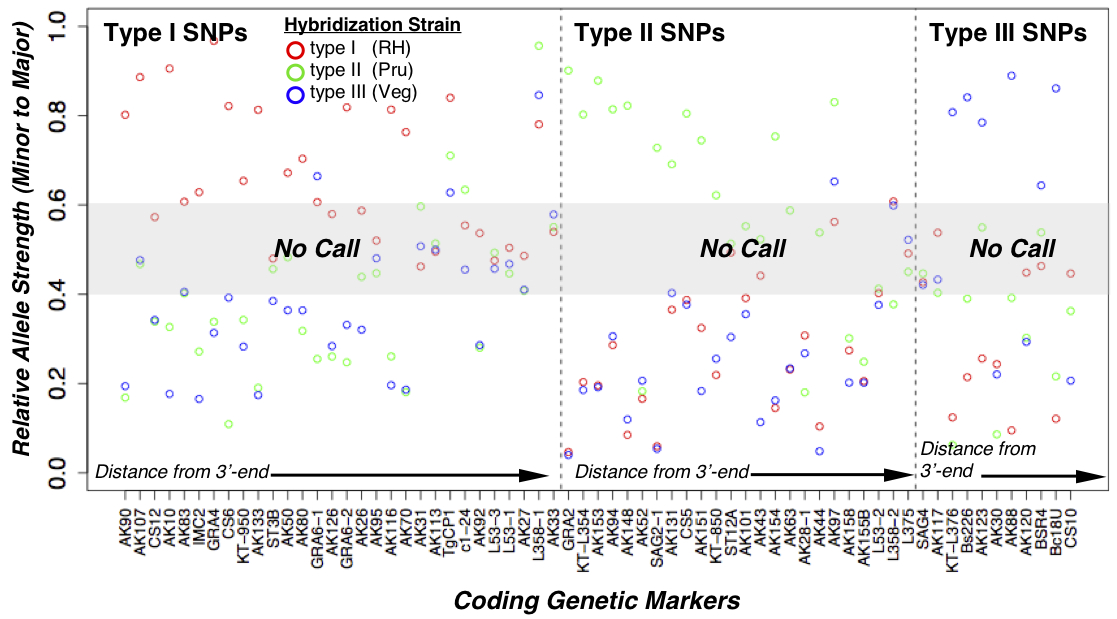

Supplement: Additional file 5 — Multiplexing experiments. The ability to reliably differentiate alleles of RFLP genetic markers that fall within coding regions using RNA hybridization data is illustrated (i.e. genotyping analysis as described in the Methods section applied to RNA hybridizations). For example, the type I RH strain correctly exhibits high relative minor allele strength (minor allele/(major allele + minor allele)) for most type I SNPs, but not for type II or type II. In addition, miscall rates are very low when the marker is close to the 3-prime end of the gene, but rise appreciably after ~1000 bp. [file 1471-2164-11-603-S5.JPEG]

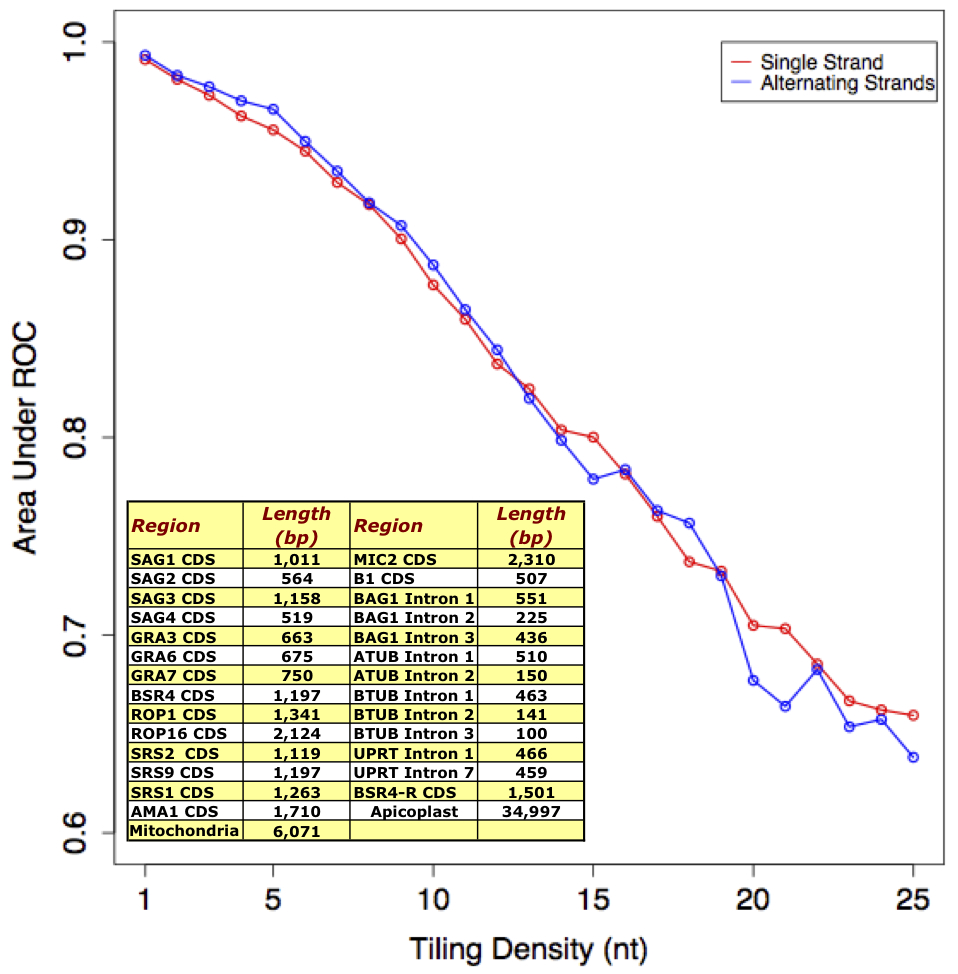

Supplement: Additional file 6 — Tiling density for SNP discovery. The ability to detect known homozygous mouse SNPs decreases with increasing distance between the centers of successive probes, as illustrated by the area under the curve (AUC) of the ROC measurements derived from a custom SNP classifier applied to each gap size. A 2-bp tiling strategy, with adjacent probes on alternate strands, offers near perfect SNP detection. The inset table lists the genomic loci that were tiled. [file 1471-2164-11-603-S6.JPEG]

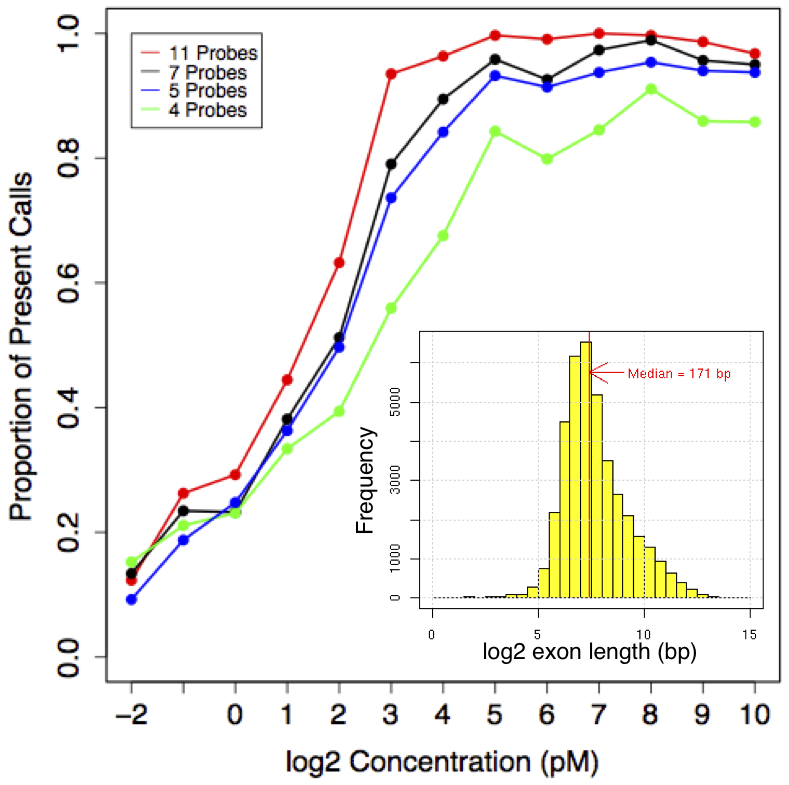

Supplement: Additional file 7 — Probe density for exon-level analysis. HGU95 spike-in data (Affymetrix) was used to test the effects of decreasing probe number on present/absent calls using the MAS5 algorithm. Five probes offer reliable transcript detection across a dynamic range ≥8 pM; as the median exon size in T. gondii is 171 bp (inset), a tiling density of 35 bp was selected for exon discovery probes. In order to err on the side of conservatism, six probes were selected for the 'all exon' probesets on chromosome Ib. [file 1471-2164-11-603-S7.JPEG]
